# The Effect of Testosterone on Serum Lipid Profiles, Glucose, Insulin and Leptin: A Experimental Study Based Animal Model

**DOI:** 10.31661/gmj.v13i.3355

**Published:** 2024-08-11

**Authors:** Adere Akhtar, Tooba Sohbatzadeh, Ramina Fazeli, Gholamhossein Ranjbar Omrani, Melika Shojaei

**Affiliations:** ^1^ Yasuj University of Medical Sciences, Yasuj, Iran; ^2^ Student Research Committee, School of medicine, Alborz University of Medical Science, Alborz, Iran; ^3^ Endocrine and Metabolism Research Center, Department of Internal Medicine, Nemazee Hospital, Shiraz University of Medical Science, Shiraz, Iran

**Keywords:** Leptin, Testosterone, Insulin, Lipid Profile, Glucose

## Abstract

Background: Disruption in the endocrine system can cause many diseases. Based on this, the imbalance of sex hormones such as testosterone can change many serum factors. In this study, we examined the effect of testosterone on leptin levels, lipid profiles, and ultimately insulin resistance.Materials and Methods: Twenty one adult rats were divided into three groups of 7, including control group (C), olive oil group (O), and olive oil and testosterone group (OT). In the O and OT groups, they received olive oil and olive oil in combination with testosterone injection at the dose of 2 mg/kg/day, respectively. To evaluate the effects of hormonal imbalance on insulin resistance, various parameters such as leptin, triglyceride, cholesterol, glucose, and insulin were assessed.Results: The results showed that Triglyceride (TG) and insulin levels were higher in the OT group compared to the other two groups (P0.05). In contrast, leptin and cholesterol levels were higher in group C compared to the other two groups (P0.05) and glucose levels were higher in group O compared to the other two groups (P=0.01). Conclusion: In general, it can be said that testosterone can change serum lipid profiles, leptin, insulin, and glucose.

## Introduction

Metabolic syndrome (MS) is an endocrine disorders that has affected many men and women in the world [1]. It is characterized by disturbances in glucose metabolism, insulin secretion, obesity, hypertension, and lipid profile imbalance [2][3][4]; these risk factors lead to an increase in the incidence of cardiovascular disease (CVD) in the MS patients. So far, the main pathogenesis of the disease has not been fully identified, however, it is known that obesity can be one of the main factors involved in the occurrence and progression of MS. Based on the evidence shown, mediators secreted from adipose tissue play an important role in the pathogenesis of MS [5].

Hormonal imbalance is one of the findings in the MS patients. At the physiological and molecular level, it can affect many macromolecules; lipid profiles, leptin, and insulin [6][7][8].

Insulin resistance is also observed in MS patients. It is affected by many factors and leads to the aggravation of clinical symptoms and progression of disease. Based on the evidence, it has been determined that adipose tissue, lipids, and sex hormones including testosterone can affect insulin resistance [9][10][11].

Leptin is one of the factors secreted by adipose tissue; its level in MS patients can be related to the incidence of insulin resistance [12]. Yun et al. showed that increased leptin levels in MS patients can be associated with CVD occurrence [13]. On the other hand, it has been shown that sex hormones such as estrogen and testosterone can affect leptin secretion [14]. Most studies have evaluated the role of testosterone on lipid profiles, leptin, blood sugar levels, and insulin resistance separately. Rao et al. They showed that testosterone deficiency in MS and diabetic patients can lead to disturbances in glucose metabolism as well as lipid oxidation [15]. Also, Wan et al. showed that there was an inverse relationship between testosterone levels and insulin resistance in male patients [16].

The difference between the present study and previous studies is in the type of study. In previous studies, testosterone serum levels were measured in patients, while in the present study, unlike previous studies, testosterone was injected into rats.

In addition, in previous studies, testosterone has been evaluated in male patients, while in the present study, testosterone injection to female rats has been evaluated in order to evaluate glucose and lipids metabolism.

## Materials and Methods

**Table:** Table1. Evaluation of Lipid Profiles, Glucose, Insulin, and Leptin between Three Groups

Variables	Group1 C	Group2 O	Group OT	P-value
TG	120.14±47.11	159.00±52.46	363.57±68.53	<0.001
Cholesterol	60.28±12.40	56.57±4.31	46.85±8.35	0.03
Glucose	112.00±21.84	142.86±15.28	128.14±14.28	0.01
Leptin	1.32±0.13	0.99±0.09	1.14±0.26	0.01
Insulin	4.70±2.26	10.41±3.73	11.81±7.61	0.03

**Table:** Table2. Evaluation of Lipid Profiles, Glucose, Insulin, and Leptin between the C and O Groups

Parameters	Control (C)		Olive Oil (O)	
		Before (B)	After (A)	P Value
TG	120.14±47.11	129.5±32.76	159±54.46	0.12
Cholesterol	60.28±12.4	58.57±5.22	56.57±4.31	0.41
Glucose	112±21.84	144.85±22.05	142.85±15.27	0.85
Leptin	1.32±0.13	1.21±0.16	0.99±0.09	0.01
Insulin	4.7±2.26	4.95±2.9	10.41±3.73	0.003

Animals

Adult rats (204.9±1.9 g and 51-54 day) were obtained under the pathogen-free conditions from Namazi Hospital Laboratory. They were individually kept in a clear polycarbonate cage under 12:12 hour light-dark photoperiod, stable ambient temperature (24 °C), and relative humidity of 50±10% for at least three weeks before and during the experimental work. All rats were kept under standard conditions and their diet was implemented according to the instructions [17]. Based on the international guidelines for working with animals, the process of evaluating the interventions and checking the desired factors was performed [17].

Drugs

To prepare solutions for injection, testosterone (testosterone enanthate, Aburaihan Company, Tehran-Iran) was dissolved in olive oil (Hojiblanka, extra virgin, Spain). The injection was done with an insulin syringe and subcutaneously (sc). The dose selection of injectable substances and the use of olive oil as a vehicle were selected based on previous studies and related sources [17][18]. The body weight and examination of each rat was recorded daily to the nearest 0.1 g, measured just prior to intervention. At first, the rats were taken out of the cage; then they were euthanized using ketamine and xylol. Finally, the blood specimen was taken from rats.

Study Design

The animals were randomly allocated into three groups:

Control group "C" (n=7): Received no injection for three weeks.

Olive oil group "O" (n=7): Received only olive oil injection (2mg/kg) for three weeks.

Olive oil and testosterone group "O"T (n=7): Received testosterone injection, diluted in olive oil (1mg/kg of olive oil with 1mg/kg of testosterone), during three weeks.

During the intervention, the animals were examined every day. Also, their storage place, food ration, and the number of animals in each shelf were based on the international guidelines.

Hormonal Measurements

Serum was used to measure hormones. For this purpose, blood samples were collected from rats and after centrifugation, serum was immediately collected and stored at -70°C. To measure leptin, the radioimmunoassay (RIA) method was used by the corresponding ELISA kit (Linco Research, St. Louis, MO, Product No.: L146) according to the relevant instructions. Also, the amount of insulin was measured using the RIA method and the relevant kit (American Laboratory Products Company (ALPCO), USA, Catalog 80-INSRT-E01). Glucose oxidase method was used to measure glucose. Colorimetric and fluorometric methods were perormed to measure Triglyceride (TG) and cholesterol, respectively [19].

Statistical Analysis

Data showed as mean ± SD. ANOVA-One way was used for the analysis of changes from baseline for each biochemical parameter. Differences between the age groups were explored by Tukey s test. The SPSS version 22 (IBM Corp., Armonk, NY., USA) used for data analysis.

Ethical Approval

This article does not contain any studies with human participants by any of the authors. We used animal model as the sample for study (Ethic ID: IR.SUMS.MED.REC.1398.623).

## Results

**Table:** Table3. Evaluation of Lipid Profiles, Glucose, Insulin, and Leptin between the C and OT Groups

**Parameters**	**Control (C)**		**Olive Oil and Testosterone (OT)**	
		**Before (B)**	**After (A)**	**P Value**
TG	120.14±47.11	128±23.06	363.57±68.53	<0.001
Cholesterol	60.28±12.4	53.14±6.30	46.85±8.35	0.41
Glucose	112±21.84	123±24.47	128.14±14.28	0.39
Leptin	1.32±0.13	1.12±0.08	1.14±0.26	0.82
Insulin	4.7±2.26	1.12±0.08	1.14±0.26	0.82

**Table:** Table4. Intergroup Comparisons

Parameters	Control (C)		Multiple Comparison P Value (For after injection)	
		OT vs O	OT vs C	O vs C
TG	120.14±47.11	<0.001	<0.001	0.21
Cholesterol	60.28±12.40	0.17	0.03	0.99
Glucose	112±21.84	0.39	0.30	0.01
Leptin	1.32±0.13	0.40	0.21	0.008

Evaluation of Lipid Profiles, Glucose, Insulin, and Leptin between the Three Groups

**Figure-1:**
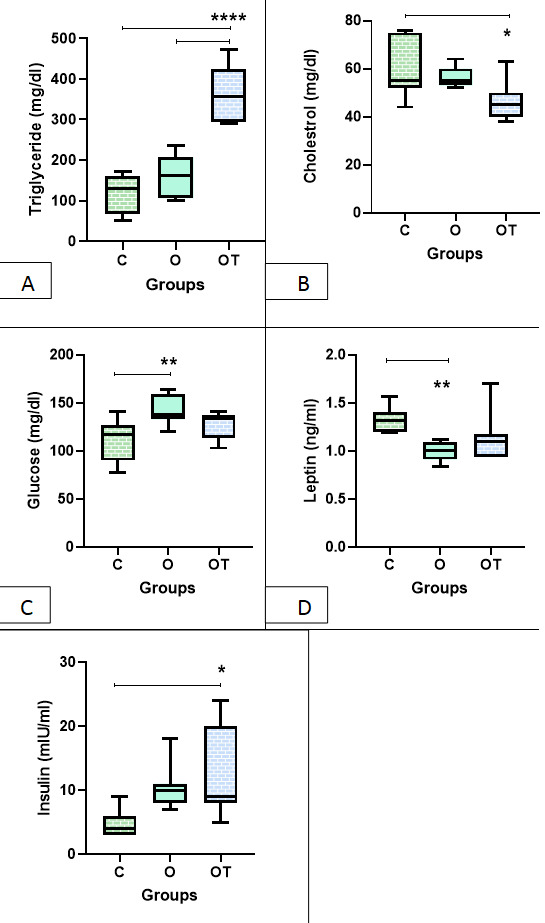
Figure 1. The figure shows the level of variables between two groups. A: TG level was higher in OT group compared to C (P<0.05). B: Cholesterol level was higher in group C compared to OT (P< 0.05). C: Glucose level was higher in group O compared to group C (P<0.05). D: leptin level was decreased in group O compared to group C (P<0.05). E: The insulin level in the OT group was higher compared to the O and C groups (P<0.05).

Based on the table below, it was shown that the average of TG and insulin in the OT group was statistical higher compared to the other two groups (P=0.03 for insulin and P<0.001 for TG). It was also found that the mean of cholesterol and leptin in group O was statistical higher compared to the other two groups (P=0.03 for cholesterol and P=0.01 for leptin). The average level of glucose in group O was statistical higher compared to the other two groups (P=0.01, Table-1, Figure-1).

Evaluation of Lipid Profiles, Glucose, Insulin, and Leptin between the C and O Groups

In the Table-2, the average of variables are shown before and after the intervention in group O in comparison with group C. The results showed that the average of TG and insulin after the intervention in group O was statistical higher compared to the pre-intervention and also group C (P=0.12 for TG and P=0.003 for insulin). The mean of leptin and cholesterol was higher in group C compared to the pre and post intervention in group O which only for leptin statistical significance (P=0.01 for leptin and P=0.41 for cholesterol). The mean glucose level was higher in group O in the pre-intervention and group C which not significant (P=0.85, Table-2).

Evaluation of Lipid Profiles, Glucose, Insulin, and Leptin between the C and OT Groups

The results showed that the average of TG and glucose after the intervention in the OT group increased, and also it was higher than the C group. However, this difference for TG statistical significance (P=0.39 for glucose and P<0.001 for TG). In contrast, the mean level of cholesterol, leptin, and insulin were higher in group C compared to the pre and post-intervention period in the OT group. However, this difference not statistical significance (P=0.41 for cholesterol, P=0.82 for leptin and insulin, Table-3).

Intergroup Comparisons

Table-4 has evaluated the variables between the three groups. The comparison of TG between OT vs O and OT vs C groups was significant (P<0.001). In relation to cholesterol, there was a significant relationship between OT vs C (P=0.03). For glucose and leptin, there was a significant relationship between O vs C groups, and for insulin between OT vs C (P=0.01 for glucose and P=0.008 for leptin, Table-4).

## Discussion

In our study, the results showed that the injection of testosterone along with olive oil increased the level of TG and glucose, and also decreased cholesterol along with insulin and leptin. On the other hand, the injection of olive oil led to an increase in the level of insulin and TG and also decreased cholesterol, glucose, and leptin.

In previous studies, there are challenging results regarding the relationship between testosterone and glucose, insulin, and lipid profiles. For this purpose, it was shown in a study that the relationship between testosterone and glucose level can be negative. Based on this, a disturbance in the hormone level can lead to an increase in blood sugar and the occurrence of diabetes in patients [20]. In another study, it was shown that hormone therapy with testosterone led to a decrease in TG, cholesterol, and HbA1C levels in patients during a one-year follow-up period [21]. Consistent with the present study, a study has reported that testosterone treatment in obese men with type 2 diabetes reduced insulin and cholesterol; but, unlike the present study, glucose and TG level reduced [22].Mentioned studies have investigated human samples. In addition, some studies were conducted on patients with diabetes or metabolic syndrome. In addition, the treatment of male patients was done using hormone therapy through testosterone injection. These cases can cause discrepancies in the results of the present study with previous ones. In the present study, female rat underwent intervention. In a series of other studies, the investigation has been carried out on animal models similar to the present study. Fillippi et al. showed that the treatment of mice with testosterone resulted in blood sugar decrement and an improvement in lipid profiles [23]. In another study, the treatment of testosterone-deficient mice led to improvement in blood sugar and prevention of insulin resistance [24]. Our findings showed a decrement in leptin levels in the two groups after the intervention. In previous studies, it was shown that testosterone, independently of leptin reduces glucose regulation and insulin levels [25]. Insulin and leptin interact with each other; leptin leads to the secretion of testosterone by regulating the expression of estrogen. In addition, due to the regulation of genes and molecular pathways, it leads to the disruption of endocrine system [26].

In summary, short term 2mg/kg/d testosterone affects lipid and insulin metabolism in young female rats. However, it is suggested to design further researches regarding the sex steroid action, age groups of rat, and changes in food chain. This study have some limitation including sample size and short term duration of follow up. We propose a long-term study lasted for more than 12 weeks and monitoring the change of serum leptin, insulin, TG, LDL(Low-density lipoprotein), and HDL(High-density lipoprotein) in rats after loss of estrogen and reexamine the influence of estrogen replacement.

## Conclusion

In general, the injection of testosterone along with olive oil increased TG and glucose and decreased cholesterol along with insulin and leptin. On the other hand, the injection of olive oil increased the level of insulin and TG, and decreased cholesterol, glucose, and leptin.

## Acknowledgment

We wish to thank all our colleagues in the shiraz university of medical science. The authors would like to thank the Metabolism and Endocrine Research Center of Shiraz University of Medical Sciences and Institute of Animal Care of Namazi Hospital.

## Conflict of Interest

The authors declare that they have no conflict of interest.

## References

[R1] Lemieux I, Després JP (2020). Metabolic Syndrome: Past, Present and Future. Nutrients.

[R2] Bovolini A, Garcia J, Andrade MA, Duarte JA (2021). Metabolic Syndrome Pathophysiology and Predisposing Factors. Int J Sports Med.

[R3] Hoe KK, Han TL, Saint Hoe (2023). Hypoglycemic agents and prognostic outcomes of chronic kidney
disease
patients with type 2 diabetes. J Nephropathol.

[R4] Salehi MR, Ghaemi M, Masoumi S, Azadnajafabad S, Norooznezhad AH, Vahdani FG, et al (2023). Comparative Analysis of Corticosteroid Therapy in Pregnant Women
with
COVID-19: Evaluating Glycemic Control and Transient Hyperglycemia. Fertility, Gynecology and Andrology.

[R5] Silveira Rossi, Barbalho SM, Reverete de, Bechara MD, Sloan KP, Sloan LA (2022). Metabolic syndrome and cardiovascular diseases: Going beyond
traditional
risk factors. Diabetes Metab Res Rev.

[R6] Chang E, Patel B (2024). Role of Hormonal Imbalance in the Pathogenesis of Metabolic
Syndrome: A
Comprehensive Review. Advances in Human Physiology Research.

[R7] Marzban M, Bahrami M, Kamalinejad M, Tahamtan M, Khavasi N, Haji M (2022). The therapeutic effects of chicory seed aqueous extract on
cardio-metabolic profile and liver enzymes in nonalcoholic fatty liver
disease; a
double blind randomized clinical trial. Immunopathol Persa.

[R8] Borna S, Ashrafzadeh M, Ghaemi M, Eshraghi N, Hivechi N, Hantoushzadeh S (2023). Correlation between PAPP-A serum levels in the first trimester of
pregnancy with the occurrence of gestational diabetes, a multicenter cohort
study. BMC Pregnancy Childbirth.

[R9] Gluvic Z, Zaric B, Resanovic I, Obradovic M, Mitrovic A, Radak D, Isenovic ER (2017). Link between Metabolic Syndrome and Insulin Resistance. Curr Vasc Pharmacol.

[R10] Aledan H, Saadi SJ, Rasheed J (2023). Evaluation of effects of glucagon-like peptide-1 receptor
agonists and
sodium-glucose co-transporter-2 inhibitors on estimated glomerular
filtration rate,
albuminuria and weight in diabetic kidney disease: A prospective cohort
study. J Renal Inj Prev.

[R11] Naeiji Z, Gargar SS, Pooransari P, Rahmati N, Mirzamoradi M, Eshraghi N, Ghaemi M, Arbabzadeh T, Masoumi M, Shamsinezhad BB, Omidi Kermanshahaninejad (2023). Association between fetal liver diameter and glycemic control in
pregnant
women with gestational diabetes: A pilot study. Diabetes Metab Syndr.

[R12] Gao Y-H, Zhao C-W, Liu B, Dong N, Ding L, Li Y-R, et al (2020). An update on the association between metabolic syndrome and
osteoarthritis and on the potential role of leptin in osteoarthritis. Cytokine.

[R13] Yun JE, Kimm H, Jo J, Jee SH (2010). Serum leptin is associated with metabolic syndrome in obese and
nonobese
Korean populations. Metabolism.

[R14] Fabian UA, Charles-Davies MA, Fasanmade AA, Olaniyi JA, Oyewole OE, Owolabi MO, et al (2016). Male Sexual Dysfunction, Leptin, Pituitary and Gonadal Hormones
in
Nigerian Males with Metabolic Syndrome and Type 2 Diabetes Mellitus. J Reprod Infertil.

[R15] Rao PM, Kelly DM, Jones TH (2013). Testosterone and insulin resistance in the metabolic syndrome and
T2DM in
men. Nat Rev Endocrinol.

[R16] Kurniawan LB, Adnan E; (2020). Insulin resistance and testosterone level in Indonesian young
adult
males. Rom J Intern Med.

[R17] Council NR, Earth Do, Studies L, Research IfLA, Care CftUotGft, Animals UoL (2010).

[R18] Zhao Z, Shi A, Wang Q, Zhou J (2019). High oleic acid peanut oil and extra virgin olive oil
supplementation
attenuate metabolic syndrome in rats by modulating the gut microbiota. Nutrients.

[R19] Gupta AK, Jain SK (2004). A study to evaluate surrogate markers of insulin resistance in
forty
euglycemic healthy subjects. J Assoc Physicians India.

[R20] Gucenmez S, Yildiz P, Donderici O, Serter R (2024). The effect of testosterone level on metabolic syndrome: a
cross-sectional
study. Hormones.

[R21] Canguven O, Talib R, El Ansari, Yassin DJ, Salman M, Al-Ansari A (2017). Testosterone therapy has positive effects on anthropometric
measures,
metabolic syndrome components (obesity, lipid profile, Diabetes Mellitus
control),
blood indices, liver enzymes, and prostate health indicators in elderly
hypogonadal
men. Andrologia.

[R22] Groti K, Žuran I, Antonič B, Foršnarič L, Pfeifer M (2018). The impact of testosterone replacement therapy on glycemic
control,
vascular function, and components of the metabolic syndrome in obese
hypogonadal men
with type 2 diabetes. Aging Male.

[R23] Filippi S, Vignozzi L, Morelli A, Chavalmane AK, Sarchielli E, Fibbi B, Saad F, Sandner P, Ruggiano P, Vannelli GB, Mannucci E, Maggi M (2009). Testosterone partially ameliorates metabolic profile and erectile
responsiveness to PDE5 inhibitors in an animal model of male metabolic
syndrome. J Sex Med.

[R24] Kelly DM, Akhtar S, Sellers DJ, Muraleedharan V, Channer KS, Jones TH (2016). Testosterone differentially regulates targets of lipid and
glucose
metabolism in liver, muscle and adipose tissues of the testicular feminised
mouse. Endocrine.

[R25] Vojnović Milutinović, Teofilović A, Veličković N, Brkljačić J, Jelača S, Djordjevic A, et al (2021). Glucocorticoid signaling and lipid metabolism disturbances in the
liver
of rats treated with 5α-dihydrotestosterone in an animal model of polycystic
ovary
syndrome. Endocrine.

[R26] Khodamoradi K, Khosravizadeh Z, Seetharam D, Mallepalli S, Farber N, Arora H (2022). The role of leptin and low testosterone in obesity. Int J Impot Res.

